# Regulation of the stem cell marker CD133 is independent of promoter hypermethylation in human epithelial differentiation and cancer

**DOI:** 10.1186/1476-4598-10-94

**Published:** 2011-07-29

**Authors:** Davide Pellacani, Richard J Packer, Fiona M Frame, Emma E Oldridge, Paul A Berry, Marie-Christine Labarthe, Michael J Stower, Matthew S Simms, Anne T Collins, Norman J Maitland

**Affiliations:** 1 YCR Cancer Research Unit, Department of Biology, University of York, Wentworth Way, York, YO10 5DD, UK; 2 Pro-Cure Therapeutics Ltd, The Biocentre, Innovation Way, York Science Park, Heslington, York, UK; 3 York District Hospital, Wigginton Road, City Centre, York, YO31 8HE, UK; 4 Castle Hill Hospital, Castle Rd, Cottingham, East Yorkshire, HU16 5JQ, UK

**Keywords:** CD133, prostate cancer, cancer stem cells, epigenetic regulation of gene expression, DNA methylation

## Abstract

**Background:**

Epigenetic control is essential for maintenance of tissue hierarchy and correct differentiation. In cancer, this hierarchical structure is altered and epigenetic control deregulated, but the relationship between these two phenomena is still unclear. CD133 is a marker for adult stem cells in various tissues and tumour types. Stem cell specificity is maintained by tight regulation of CD133 expression at both transcriptional and post-translational levels. In this study we investigated the role of epigenetic regulation of CD133 in epithelial differentiation and cancer.

**Methods:**

DNA methylation analysis of the CD133 promoter was done by pyrosequencing and methylation specific PCR; qRT-PCR was used to measure CD133 expression and chromatin structure was determined by ChIP. Cells were treated with DNA demethylating agents and HDAC inhibitors. All the experiments were carried out in both cell lines and primary samples.

**Results:**

We found that CD133 expression is repressed by DNA methylation in the majority of prostate epithelial cell lines examined, where the promoter is heavily CpG hypermethylated, whereas in primary prostate cancer and benign prostatic hyperplasia, low levels of DNA methylation, accompanied by low levels of mRNA, were found. Moreover, differential methylation of CD133 was absent from both benign or malignant CD133^+^/α_2_β_1_integrin^hi ^prostate (stem) cells, when compared to CD133^-^/α_2_β_1_integrin^hi ^(transit amplifying) cells or CD133^-^/α_2_β_1_integrin^low ^(basal committed) cells, selected from primary epithelial cultures. Condensed chromatin was associated with CD133 downregulation in all of the cell lines, and treatment with HDAC inhibitors resulted in CD133 re-expression in both cell lines and primary samples.

**Conclusions:**

CD133 is tightly regulated by DNA methylation only in cell lines, where promoter methylation and gene expression inversely correlate. This highlights the crucial choice of cell model systems when studying epigenetic control in cancer biology and stem cell biology. Significantly, in both benign and malignant prostate primary tissues, regulation of CD133 is independent of DNA methylation, but is under the dynamic control of chromatin condensation. This indicates that CD133 expression is not altered in prostate cancer and it is consistent with an important role for CD133 in the maintenance of the hierarchical cell differentiation patterns in cancer.

## Background

Epigenetic regulation of gene expression is a dynamic mechanism, which permits precise regulation throughout differentiation [1]. It plays a crucial role in preserving the hierarchical structure of tissues and is involved in maintaining stemness and fate determination of adult stem cells [2,3]. Indeed, DNA methylation varies throughout cell differentiation [4] and epigenetic control is required for the multipotency of hematopoietic stem cells [5].

There is mounting evidence to support the hypothesis that cancers can retain the hierarchical structure present in normal tissues, but that homeostasis is disrupted, leading to aberrant replication and differentiation. As in normal tissues, a small percentage of cancer cells can persist and initiate new tumour growth (tumour-initiating cells or cancer stem cells - CSCs), while most cells proceed to terminal differentiation [6].

CD133 is a pentaspan membrane glycoprotein first identified in humans as a hematopoietic stem cell marker [7] and is currently used for the identification of stem cells from several tissues and cancer types [8]. In non-malignant human prostate, CD133 and α_2_β_1_integrin are two markers that, when used in combination, have been demonstrated to enrich for a cell population with stem cell features [9]. Indeed, CD133^+ ^cells from human primary tissues represent a very small subpopulation (0.1-3.0% of the total prostatic epithelium) located in the basal compartment. Tissue stem cells are restricted to the α_2_β_1_integrin^hi ^population, have a high colony-forming ability and proliferative potential, and are able to regenerate fully differentiated prostate epithelium in vivo. The stem cells express basal cell markers, such as CD44 and CK5/14, but do not express luminal markers AR, PSA or depend on androgens for their survival [9-11]. Other markers have been used to identify prostate stem cells, for example, in murine models, where selection markers include Sca-1 [12] and CD117 [13].

CD133^+ ^cells from prostate cancer biopsies (PCSCs) are similar in phenotype to normal prostate stem cells. They are rare (0.1-0.5% of the total cell population) and represent the clonogenic population with highest proliferative potential. Moreover, they express basal cell cytokeratins, but do not express AR [14].

Disruption of epigenetic mechanisms is found in all cancers and, together with genetic changes, plays a key role in cancer initiation and progression [15]. Epigenetic studies in prostate cancer (CaP) have resulted in the identification of hundreds of hypermethylated genes, of which GSTP1 is the most studied [16], as well as changes to chromatin structure and histone-modifying enzymes [15].

However, these studies do not take into account the hierarchical structure of cancer, since they describe epigenetic alterations that occur in the bulk population of cancer cells. It has been proposed that disruption of epigenetic control may result in formation of aberrant self-renewing cells [17], culminating in a complete deregulation of the hierarchical system, ultimately leading to cancer [18]. Thus, understanding how epigenetic regulation of gene expression controls the differentiation process and its deregulation in cancer is of great importance in order to develop new therapeutic strategies for cancer, directed to the therapy resistant cancer stem cell population.

**Figure 1:**
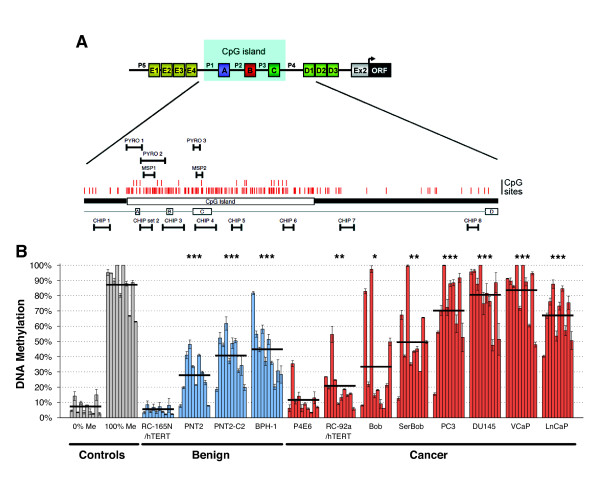
**CD133 promoter is hypermethylated in prostate cancer cell lines**. (A) The CD133 promoter (adapted from Shmelkov et al., 2004 [20]) (A,B,C,D1-3, E1-4 = alternative first exons; P1-P5 = promoters; Ex2 = Exon 2; ORF = open reading frame; PYRO 1-3: pyrosequencing assays; MSP1-2: MSP assays; CHIP 1-8: ChIP primers). (B) Pyrosequencing analysis of the CD133 promoter performed in prostate cell lines (PYRO 1 assay; bars = single CpG sites; n = 3 technical replicas;  ± SD; black line = average of 10 CpG sites; * = p < 0.05; ** = p < 0.01; *** = p < 0.001 in an unpaired t-test).

CD133 expression is controlled at multiple steps including transcriptional regulation, alternative transcription initiation sites, alternative splicing and post-translational modifications [8,19]. This fine regulation results in the maintenance of stem cell-specific CD133 expression patterns. Twelve different mRNA isoforms, generated by alternative splicing, have been described in various mammals (CD133.s1 - s12), of which at least seven are expressed in human cells [19]. Moreover, five different alternative first exons, regulated by five TATA-less promoters, have been described [20] (Figure 1A). Transcription is initiated from different first exons in a tissue specific manner. In particular, only exon 1A is expressed in prostate, indicative of promoter P1 activity. The presence of a large CpG island in the CD133 promoter area and the silencing of promoters P1 and P2 transcriptional activity by in vitro DNA methylation, suggest that this gene can be regulated by epigenetic mechanisms [20].

Here, we show that CD133 expression is efficiently repressed by promoter methylation in prostate cell lines. However, in primary cultured cells from prostate epithelium and tumour xenografts, regulation of CD133 expression is independent of DNA methylation, indicating that, for this gene, cell lines are not indicative of DNA methylation status in primary tissues. In addition, the CD133 promoter is not hypermethylated in prostate cancer tissues, highlighting the important role for CD133 in the maintenance of the hierarchical structure of cancer.

## Results

### The CD133 promoter region is hypermethylated in prostate cancer cell lines

CD133 promoter methylation was quantified by pyrosequencing in prostate cell lines (Figure 1B, Additional File 1, Figure S1B-C) using 3 assays (PYRO 1-3 assays) located on promoter P1/exon 1A, promoter P2 and exon 1C respectively (Figure 1A). Using the PYRO 1 assay, significant hypermethylation was found in the majority of cell lines analysed, relative to the 0% methylation control (0% Me) (p < 0.05), apart from the benign cell line RC-165N/hTERT and the cancer cell line P4E6 (Figure 1B). However, when each individual CpG site was analysed separately, significant hypermethylation (p < 0.05) was found at CpG sites 2-3-4 in P4E6 cells. Very high levels of CD133 promoter methylation were found in the CaP cell lines PC3 (70%), DU145 (80%), VCaP (83%) and LnCaP (67%) (p < 0.001). The benign cell lines BPH-1, PNT2 and PNT2-C2, also showed significant hypermethylation (p < 0.001 - average methylation between 30% and 50%). The CaP-derived Bob and SerBob cell lines showed a very heterogeneous pattern of methylation throughout the sequence analysed, with higher levels of methylation in SerBob (p < 0.01) than Bob (p < 0.05). Significant, but low methylation levels (p < 0.01) were found in the RC-92a/hTERT cancer cell line.

To assess whether the levels of methylation were consistent along the entire CpG island, PYRO 2 and 3 assays were carried out, and both showed comparable patterns of methylation with the PYRO 1 assay (Additional File 1, Figure S1B-C). Finally, to confirm our findings, a standard methylation-specific PCR (MSP) was also carried out (Additional File 1, Figure S1A) and gave results that matched with those obtained by pyrosequencing.

The data summarised in Figure 1B implies that both malignancy and culture conditions (presence of fetal calf serum - FCS) influences the methylation status of the CD133 CpG island. The cell lines analysed could be divided into four groups, depending on the type of tissue from which they were derived (benign or CaP) and the amount of FCS present in the culture medium (≤ 2% or >2%). A significant difference (p < 0.01) in average CD133 promoter methylation was found between benign and cancer derived cell lines cultured in high levels of FCS, but also between CaP cell lines cultured in low or high levels of FCS (Additional File 1, Figure S1D).

### CD133 expression is regulated by DNA methylation in prostate cell lines

**Figure 2:**
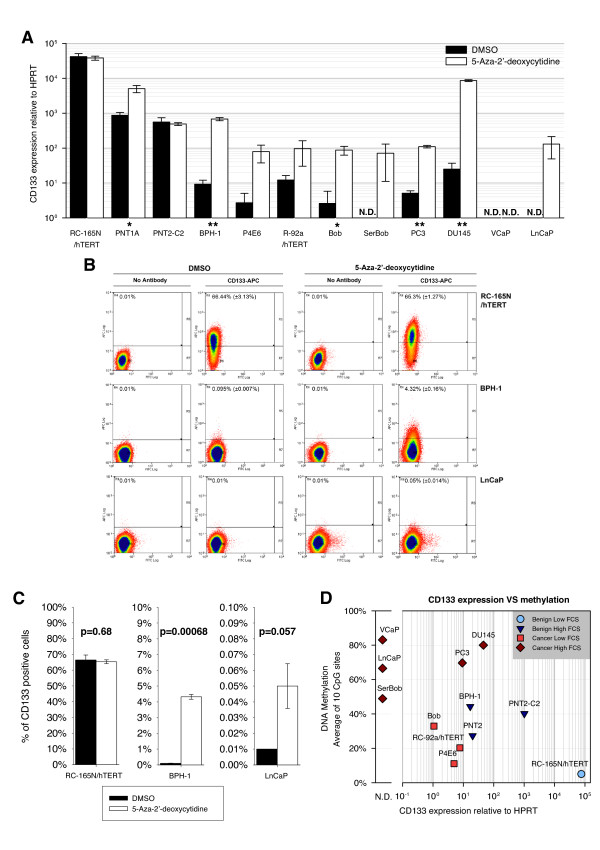
**CD133 expression is repressed by DNA methylation in prostate cancer cell lines**. (A) qRT-PCR analysis (relative to HPRT, calibrator = P4E6) of CD133 expression in prostate cell lines treated with 1 μM 5-Aza-2'-deoxycytidine for 96 hours. (N.D. = expression undetectable after 40 cycles, n = 2;  ± SD, * = p < 0.05; ** = p < 0.01 in a paired t-test, p-value not calculated in cell lines with non-detectable levels of CD133). (B, C) FACs analysis of CD133 expression in prostate cell lines treated with 1 μM 5-Aza-2'-deoxycytidine for 96 hours. Live cells were stained with CD133/2(293C)-APC antibody (Miltenyi Biotec) (CD133-APC) or without any antibody (No Antibody) and analysed by FACs. For each dot plot, X axes: FITC channel fluorescence (not stained); Y axes: APC channel fluorescence (CD133 or No Antibody control); Percent of CD133^+ ^cells is indicated and the results are summarised in C. (D) Dot-plot showing expression (qRT-PCR, relative to HPRT, calibrator = Bob) and methylation of CD133 in a panel of prostate cell lines grouped on the basis of their origin (benign or cancer) and their culture conditions (low FCS = 0%-2%; high FCS = 5%-10%).

In order to test whether CD133 expression was directly regulated by DNA methylation, prostate cell lines were treated with the demethylating agent 5-Aza-2'-deoxycytidine (1 μM for 96 h). CD133 expression (measured by qRT-PCR) was induced in 2 out of 4 benign cell lines (BPH-1 and PNT1A) and all of the CaP cell lines analysed (P4E6, RC-92a, Bob, SerBob, PC3, DU145 and LnCaP) with the exception of VCaP (Figure 2A). The lack of induction in VCaP, could be explained by the fact that this cell line has a doubling time of 5-6 days [21], which is insufficient time for the 5-Aza-2'-deoxycytidine to be incorporated into the genome during the 96 h treatment and to exploit its demethylating function. Importantly, DNA demethylation did not induce CD133 expression in RC-165N/hTERT, confirming that CD133 is not repressed by DNA methylation in this cell line.

Next, 3 cell lines representative of the panel used were treated with 1 μM 5-Aza-2'-deoxycytidine for 96 h and analysed by FACs for the expression of the glycosylated form of CD133 (Figure 2B and 2C). As expected, no significant change in CD133 expression was seen in RC-165n/hTERT after demethylation, while a significant increase was seen in BPH-1 and marked increase (although not significant) in LnCaP.

Taken together, these results show that DNA demethylation induces CD133 upregulation, indicating that promoter methylation suppresses CD133 expression in prostate cell lines.

A direct comparison between CD133 expression, measured by qRT-PCR, and DNA methylation (PYRO 1 assay), confirmed that hypermethylation of the CD133 promoter results in downregulation of gene expression (Figure 2D). Cell lines expressing high levels of mRNA had the lowest levels of promoter methylation (e.g. RC-165N/hTERT), while CD133 was strongly downregulated when high levels of methylation were present (e.g. VCaP and LnCaP). However, in cancer cells grown in low levels of FCS, the CD133 promoter showed low DNA methylation (less than 20%) associated with low, but detectable expression (P4E6 and RC-92a/hTERT). This confirmed that culture conditions influence DNA methylation of the CD133 promoter and suggests that other mechanisms, in addition to DNA methylation, resulted in downregulation of CD133 expression.

Moreover, mRNA abundance and expression of the glycosylated isoform of CD133 (detected by the 293C3 antibody) directly correlated in prostate cell lines (Additional File 2, Figure S2). All the benign cell lines (RC-165N/hTERT, PNT1A, PNT2-C2, BPH-1) and 3 of the cancer cell lines (Bob, P4E6 and RC-92a/hTERT), analysed for CD133 expression (by flow cytometry), contained CD133^+ ^cells and had detectable levels of mRNA. In contrast, CD133^+ ^cells were not found in PC3 and DU145 cells, although detectable levels of mRNA were found; while in LnCaP and VCaP cells, CD133 was not detectable at either the mRNA or protein level. Finally, in SerBob cells, although CD133 was undetectable at the mRNA level, we detected a very small subpopulation of cells expressing very low levels of CD133 protein.

### Low levels of CD133 promoter methylation are found in prostate tissues and primary epithelial cultures

**Figure 3:**
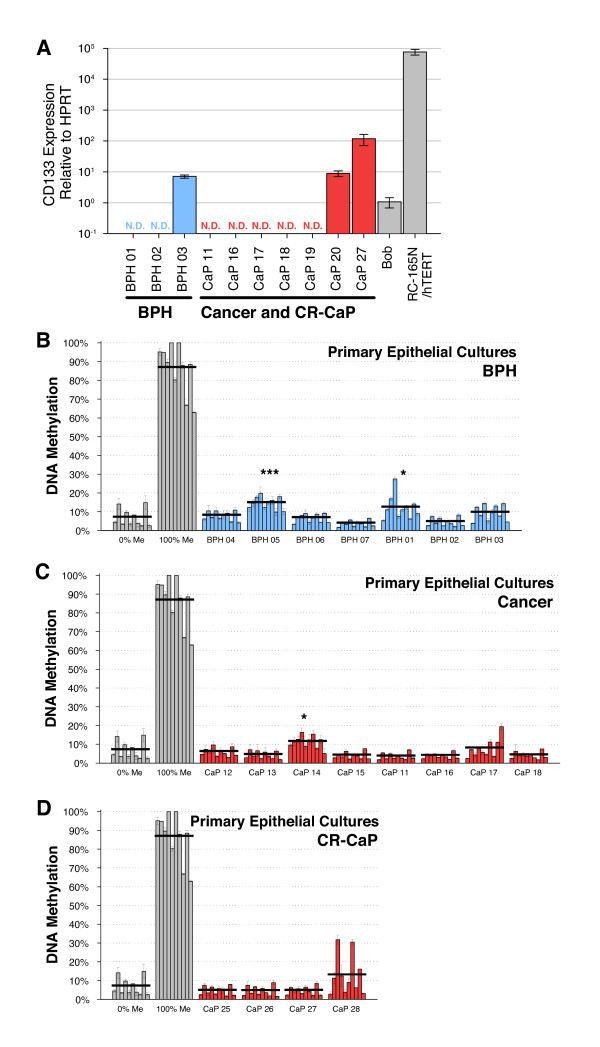
**CD133 expression and methylation in prostate primary epithelial cultures**. (A) qRT-PCR analysis (relative to HPRT, calibrator = Bob; N.D. = expression undetectable after 40 cycles) of CD133 expression in prostate primary epithelial cultures (RC-165N/hTERT and Bob were used as controls). Methylation analysis of the CD133 promoter in prostate primary epithelial cultures derived from BPH (B), CaP (C) and CR-CaP (D) by pyrosequencing (PYRO 1 assay; bars = single CpG sites; n = 3 technical replicas;  ± SD; black line = average of 10 CpG sites; * = p < 0.05; ** = p < 0.01; *** = p < 0.001 in an unpaired t-test).

CD133 expression (qRT-PCR - Figure 3A) and methylation of its promoter (PYRO 1 assay - Figure 3B-D) were then measured in primary epithelial cultures derived from clinical samples of benign prostatic hyperplasia (BPH), CaP and castration resistant prostate cancer (CR-CaP). Expression of CD133 was undetectable in 7 out of 10 samples, and detectable, but at low levels, in the remaining 3. No differences in CD133 mRNA expression were seen between BPH and CaP. These results are in line with previous results showing that CD133 is expressed (at the protein level) only in a small subpopulation of the prostate primary epithelial cultures [9,14,22,23]. DNA methylation levels were unexpectedly low, with the average methylation less than 20%, in all the samples analysed. No significant distinction was seen between BPH, CaP or CR-CaP. In fact, only 2/6 BPH, 1/8 CaP and none of the CR-CaP samples contained significantly hypermethylated DNA compared to the 0%Me control. CaP 17 (Figure 3C) and CaP 28 (Figure 3D) clearly showed hypermethylation of a few CpG sites, and separate analysis of each individual CpG site revealed significant hypermethylation (p < 0.05) in CpG site 10 for CaP 17 and sites 3 and 7 for CaP 28. The results obtained were then confirmed by pyrosequencing using the PYRO 2 assay (Additional File 3, Figure S3E) and by MSP (Additional File 3, Figure S3A). The results indicate that CD133 is not tightly regulated by DNA methylation in prostate primary epithelial cells, which was confirmed by a lack of a strong and consistent increase in CD133 expression after treatment with 5-Aza-2'-deoxycytidine (1 μM for 96 h - Not shown).

**Figure 4:**
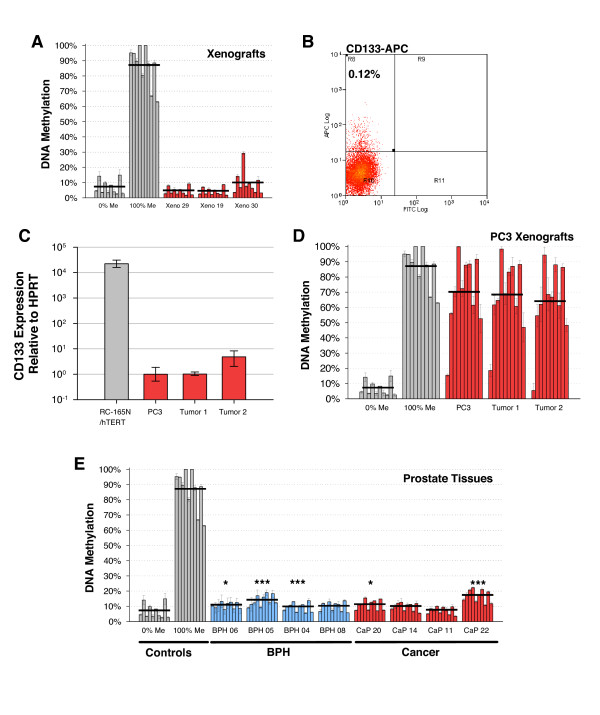
**Methylation of the CD133 promoter in prostate primary tissues and xenografts**. Methylation analysis of the CD133 promoter in primary prostate cancer xenografts generated in RAG2^-/- ^gamma C^-/- ^mice (A), in PC3 xenografts (D) and in prostate tissues (DNA extracted from glass slides of pooled snap frozen tissue sections) (E) and by pyrosequencing (PYRO 1 assay; bars = single CpG sites; n = 3 technical replicas;  ± SD; black line = average of 10 CpG sites; * = p < 0.05; ** = p < 0.01; *** = p < 0.001 in an unpaired t-test). (B) Representative image of CD133 expression measured by FACs in disaggregated primary prostate cancer xenografts. X axes: FITC channel fluorescence (not stained); Y axes: APC channel fluorescence (CD133-APC); Percent of CD133^+ ^cells is indicated. (D) qRT-PCR analysis (relative to RPLP0, calibrator = PC3; N.D. = expression undetectable after 40 cycles) of CD133 expression in PC3 xenografts compared to PC3 (RC-165N/hTERT was used as a positive control).

Lack of hypermethylation of the CD133 promoter in CaP was then confirmed in CaP xenografts recently established in RAG2^-/- ^gamma C^-/- ^immunocompromised mice (Figure 4A). None of the samples showed any significant hypermethylation of the CpG island, although Xeno 30 showed hypermethylation of CpG sites 3 and 4 (p < 0.05). Moreover, a small subpopulation of CD133 positive cells, reminiscent of the stem-like cells present in prostate cancer primary epithelial cultures [14], was present in these xenografts (Figure 4B).

To test whether the methylation of CD133 promoter is a reversible event in vivo, CD133 mRNA expression and promoter methylation was measured in xenografts generated by subcutaneous injection of PC3 cells (Figure 4C and 4D). In both the samples tested, no significant changes in expression or methylation of CD133 were found when comparing PC3 cells in vitro and in vivo. These results suggest that, once established, methylation patterns within CD133 promoter are stable and not easily reversible.

Lastly, these results were also confirmed by methylation analysis of the CD133 promoter in snap frozen prostate tissues from both benign and CaP samples (Figure 4E, Additional File 3, Figure S3C-D). 2/4 BPH samples and 2/4 CaP samples showed significantly elevated, but still very low levels (7% - 16%) of hypermethylation (p < 0.05).

### CD133 expression is not regulated by DNA methylation in the prostate epithelial hierarchy

**Figure 5:**
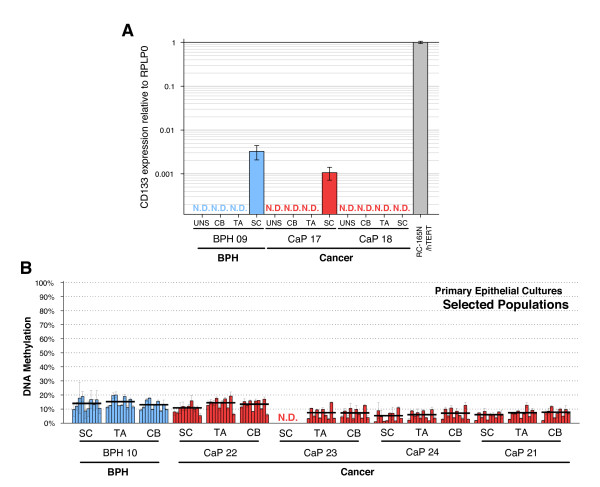
**CD133 expression and methylation in prostate hierarchy**. (A) qRT-PCR analysis of CD133 expression (relative to RPLP0, calibrator cells = RC-165N/hTERT; N.D. = expression undetectable after 40 cycles) and (B) pyrosequencing methylation analysis of the CD133 promoter in selected populations from prostate primary epithelial cultures (SC = stem cells; TA = transit amplifying cells; CB = committed basal cells: PYRO 1 assay; grey bars = single CpG sites; n = 3 technical replicas;  ± SD; the black line = average of 10 CpG sites).

CD133 expression was next analysed by qRT-PCR in SC (α_2_β_1_^hi^/CD133^+^), TA (α_2_β_1_^hi^/CD133^-^) and CB (α_2_β_1_^low^/CD133^-^) cell populations isolated from low passage (<10) primary prostate epithelial cultures (1 BPH and 2 CaP) (Figure 5A). In BPH 09 and CaP 17, CD133 expression was undetectable in unselected cultures, CB cells and TA cells, but was detectable (at low levels) in SCs. CD133 expression was not detectable, in any subpopulation, from culture CaP 18. These results provided evidence that CD133 can be regulated at the transcriptional level during differentiation of prostate epithelia from tissues.

We then wanted to analyse whether this differential regulation was sustained by DNA methylation. Pyrosequencing (PYRO 1 assay - Figure 5B) and MSP (Additional File 3, Figure S3B) were carried out on SC, TA and CB cells. Low levels of methylation (<20%) were found in all the populations. No significant differences were found between the 3 populations analysed.

### Chromatin structure, together with DNA hypermethylation, lead to CD133 downregulation in prostate epithelial cells

**Figure 6:**
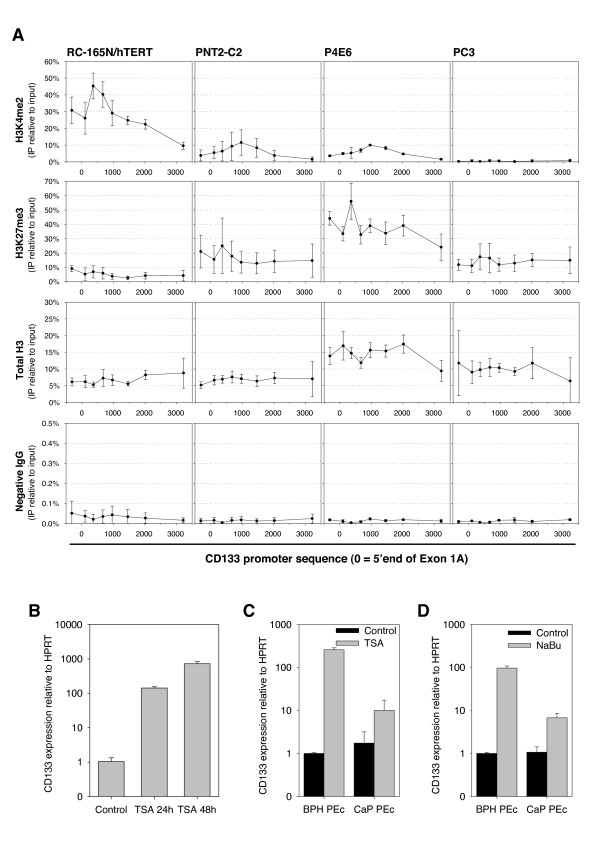
**CD133 expression is regulated by chromatin structure in prostate cell lines and primary epithelial cultures**. (A) ChIP-qPCR analysis carried out in RC-165N/hTERT, PNT2-C2, P4E6, PC3 with rabbit IgG, anti-histone H3, anti-H3K4me2, and anti-H3K27me3. qPCR primer positions are shown in Figure 1A (X axes: CD133 promoter sequence, 0 = 5'end of exon 1A; Y axes: percentage of immunoprecipitated DNA relative to input DNA; n = 3;  ± SD, data shown on logarithmic scale). (B) qRT-PCR analysis of CD133 expression in P4E6 cells lines with 0.6 μM TSA for 24 and 48 hours (relative to HPRT, calibrator = Control,  ± SD). (C,D) qRT-PCR analysis of CD133 expression in prostate primary epithelial cultures (PEc) treated with 0.6 μM TSA (C) or 10 mM NaBu (D) for 48 hours (relative to HPRT, calibrator = Control,  ± SD, data shown on logarithmic scale).

The presence of active (as detected by dimethylation of lysine 4 of histone H3 - H3K4me2) or inactive (trimethylation of lysine 27 of histone H3 - H3K27me3) chromatin around the CD133 promoter was determined by chromatin immunoprecipitation (ChIP) in RC-165N/hTERT, PNT2-C2, P4E6 and PC3 cells (Figure 6A). H3K4 dimethylation was detected in the CD133 promoter of RC-165N/hTERT cells, in accordance with the transcriptional activity. The inactive chromatin mark H3K27me3 was overrepresented in P4E6 cells, indicating that chromatin structure, rather than DNA methylation, played a more crucial role in repressing CD133 expression in this cell line. PC3 cells also showed an inactive state of chromatin, with tri-methylation of H3K27 and no enrichment for H3K4me2. PNT2-C2 showed an intermediate state of chromatin condensation where both markers for active and inactive chromatin were present, matching both the intermediate levels of methylation (around 40%) and gene expression (Figure 2A). Taken together, these data indicated that changes in chromatin structure alone, or in co-operation with DNA methylation, could result in the repression of CD133 expression.

P4E6 cells showed a very similar expression and DNA methylation pattern to primary epithelial cultures and primary tissues, containing low levels of CD133 mRNA and promoter methylation. In this cell line CD133 was maintained in a repressed state by a highly condensed chromatin structure. In line with these results, CD133 mRNA was highly overexpressed after treatment of P4E6 cells with trichostatin A (TSA, 0.6 μM for 24 and 48 hours) (Figure 6B), a well characterised histone deacetylase (HDAC) inhibitor that relaxes chromatin by inducing acetylation of histones.

Treatment of BPH and CaP derived primary epithelial cultures with 0.6 μM TSA and 10 mM NaBu (a less potent and specific HDAC inhibitor) also resulted in overexpression of CD133 mRNA (Figure 6C-D) after 48 hours, in agreement with a clear role for condensed chromatin structure in maintaining CD133 repression in both cell lines and primary samples.

## Discussion

CD133 is widely used as a marker for CSCs in many different solid tumours including: colon [24,25], brain [26,27], skin [28], pancreatic [29], liver [30,31], and prostate [14]. In both normal and cancerous prostate, the expression of CD133 is restricted to a very small subpopulation of cells with stem-like features, suggesting a tight regulation for the expression of this gene [9,14,32].

The data presented here indicated that DNA methylation mediated suppression of CD133 expression in prostate epithelial cell lines, where an inverse correlation between expression and DNA methylation was clearly seen, together with re-expression of both the mRNA and the glycosylated protein after gene demethylation by 5-Aza-2'-deoxycytidine treatment.

However, data obtained from prostate tissue and primary epithelial cultures displayed a stark contrast to that obtained in cell lines. A huge variation (spanning nearly 100,000 fold) in CD133 expression was found across the cell lines analysed, while CD133 was undetectable or expressed at very low levels in prostate primary epithelial cultures. These results provide strong evidence that regulation of CD133 expression can be disrupted during long-term culture in vitro.

Moreover, the low levels of CD133 mRNA detected in primary epithelial cultures suggested that the CD133 gene was repressed in the vast majority of prostate basal epithelial cells (with the exception of the stem cell population). The very low levels of promoter methylation found in these samples indicated that repression of CD133 expression was independent of promoter methylation and implied that other mechanisms were required to control CD133 expression in prostate tissues and primary cultures. Interestingly, CD133 expression was also not deregulated in primary prostate cancer, suggesting that a tight regulation of CD133 expression was important in the hierarchical structure of both normal and cancerous prostate.

Prostate epithelium is heterogeneous and composed of various cell populations at different differentiation stages. When SC (CD133^+^), TA and CB (CD133^-^) cell populations from primary epithelial cultures were analysed separately, the results obtained showed no differential methylation of the CD133 promoter in individual populations. Therefore mechanisms other than DNA methylation must regulate CD133 expression in the prostate epithelial cell hierarchy.

Our results indicated that one such mechanism is chromatin condensation. In prostate cell lines, a condensed status correlated with low levels of mRNA (PC3 and P4E6 cells). This is in line with the concept of crosstalk between different epigenetic mechanisms [33] and with previous findings in glioblastoma and colon cancer cell lines [34] where CD133 was regulated by both DNA methylation and histone modifications. Interestingly, chromatin structure seemed not only to parallel DNA methylation in repressing CD133 expression, but also to have an active role in repressing transcription even when hypermethylation was not present (P4E6 cells). The same mechanism was also present in primary epithelial cultures from both BPH and CaP, clearly indicating an important role for chromatin structure in repressing CD133 expression in primary prostate.

In prostate tissues, the glyscosylated form of CD133 was expressed only in a very small subpopulation of basal epithelial cells [9,35]. However, it has recently been shown that an isoform of CD133 protein that does not have the same glycosylation pattern is present in terminally differentiated prostate luminal cells [35]. So, it is clear that the CD133 gene needs to be dynamically regulated throughout differentiation of prostate epithelia, clearly supporting the hypothesis that long-term transcriptional silencing caused by DNA methylation is unlikely to affect CD133 expression in prostate epithelia. This also supports our findings that more dynamic mechanisms, such as changes in histone modifications and chromatin structure are the more likely control mechanisms.

Although CD133 is widely used as a stem cell marker in various types of cancer, very little is known about its molecular function and its functional involvement in tumour and metastasis formation. Even if there is evidence that CD133- positive cells from various cancers are more resistant to anti-cancer therapies [36-38], it is not known whether CD133 has a primary role in this resistance or it just happens to mark resistant cells. Indeed CD133 has been shown to be involved in maintaining neuroblastoma cells in an undifferentiated state, and downregulation of CD133 led to inhibition of tumour formation [39]. However, the wide expression of this surface marker in various human tissues [40] and the sparse knowledge of its molecular function pose great difficulties in using CD133 as a target for cancer stem cell therapy.

The results obtained show two different mechanisms for regulation of CD133 expression in cell lines (DNA methylation dependent) and primary tissues and recently established prostate cancer xenografts (DNA methylation independent). Although discrepancies between cell lines and primary samples have been discussed in the literature for at least 20 years [41], cell lines remain the most frequently used model for epigenetics and cancer epigenetics studies; in many cases with weak correlations to the original tissue/cancer. It is known that *de novo *methylation is a common event during cell line establishment [42,43] as part of the adaptation process that cells undergo during long-term culture. This process results in downregulation of genes that are nonessential in culture, including many tissue-specific genes [41]. The results presented here for the expression of CD133, a common stem cell marker in multiple tissues, emphasise that cell lines do not represent a valid model for DNA methylation studies, since culture conditions influence promoter methylation.

Thus, the choice of the correct cell model system is of paramount importance when studying epigenetic regulation of gene expression in cancer biology and stem cell biology. The results with CD133 highlight the need for verification (using primary tissues) of the results obtained with cell lines. For example, in the last few years, several high-throughput epigenetic studies compared commercially available normal epithelial cultures (non immortalised and cultured for a limited number of passages) with established cancer cell lines in prostate [44,45] and other tissues. In these comparisons, epigenetic adaptation of cell lines to culture conditions was not taken into account and the results obtained might be biased by *in vitro *adaptation.

Finally, in recent studies designed to isolate cells with stem cell features from prostate cancer cell lines a great discordance was reported regarding the expression of CD133 on the surface of several CaP cell lines. The data presented here, reveal the limitations of the use of cell lines in such studies, and indicate that prolonged *in vitro *culture affects the fine gene regulation that is essential for the maintenance of prostate epithelial hierarchy. Moreover, the data presented by Pfeiffer and Schalken (2010) [46], is in accordance with our data, confirming the lack of cell surface expression of CD133 in many established prostate cancer cell lines, and when expressed, CD133 did not appear to select for cells with stem cell characteristics. Our results now provide a mechanistic explanation for the apparently contrasting results presented in that study.

## Conclusions

We present here a comprehensive study of the epigenetic regulation of CD133 promoter in cell lines, primary epithelial cultures, tissue and tumour xenografts from the human prostate. We conclude that CD133 expression is regulated by different mechanisms in cell lines relative to the other samples, and that regulation in primary cultures is independent of methylation, where this gene is maintained in a repressed state by condensed chromatin structure. These results also have implications in the choice of models that are chosen to analyse epigenetic changes in cancer cells, and highlight the complexity of regulation of this common stem cell marker.

## Materials and methods

### Cell lines, tissue processing, primary epithelial cell culture and xenografts

A list of the cell lines used, origin, culture conditions and identification is provided in Additional File 4, Table S1.

Human prostatic tissue was obtained from patients undergoing transurethral and retropubic prostatectomy for BPH or undergoing radical prostatectomy for CaP (with patient consent and full ethical permission from York District Hospital and Castlehill Hospital, Hull). BPH or CaP diagnosis was confirmed by histological examination of representative adjacent fragments.

Tissues were disaggregated and cultured as described previously [9,10]. Basal cells were then cultured and further fractionated on the basis of adhesion to type I collagen [10]. CD133^+ ^cells were selected from cells that adhered within 20 minutes using MACS microbeads linked to anti-human CD133, according to the manufacturer's instruction (Miltenyi Biotec) [14].

Xenografts were generated by subcutaneous grafting of CaP tissue in RAG2^-/- ^gamma C^-/- ^mice. Tumours generated were serially passaged in vivo and routinely genotyped to confirm the original patient's genotype. Early passages (1-3) were used for DNA methylation studies.

PC3 xenografts were generated by injecting subcutaneously 106 PC3 cells embedded in 100 μl of Matrigel (BD Biosciences) in Balb/c Nude mice. Tumours were harvested after 29 days from the injection.

### DNA purification and sodium bisulfite conversion

DNA was extracted using the DNeasy Blood & Tissue Kit (Qiagen) and the QIAamp DNA micro Kit (Qiagen) for small samples. 0% and 100% methylated controls were purchased from Qiagen (EpiTect PCR Control DNA). 50 ng/1 μg of DNA was bisulfite converted using the EpiTect Bisulfite Kit (Qiagen). Converted DNA from SCs was amplified using the EpiTect Whole Bisulfitome Amplification Kit (Qiagen).

### Pyrosequencing assay

The CD133 promoter sequences were amplified by PCR using specific primers for 3 regions of the CpG island (Additional File 5, Table S2 and Figure 1A) and sequenced using the PyroMark Q24 System (Qiagen). Data were analysed with PyroMark Q24 software. In primary epithelial cultures and xenografts, a mixture of human and mouse DNA was analysed. PYRO 1 (Additional File 6, Figure S4) and PYRO 2 (not shown) assays were human specific. PYRO 3 gave non-specific PCR products with mouse DNA (not shown) so it was not used for mixed (human/mouse) samples.

### Methylation-specific PCR

Methylation-specific PCR was carried out as previously described [47] in a final 25 μL reaction mixture using 10 ng of bisulfite converted DNA as template and Platinum Taq DNA Polymerase (Invitrogen). Primer sequences are listed in Supplementary Table 2. The PCR program was: 94°C for 2 minutes, then 35 cycles of 94°C for 20 seconds, 55°C for 20 seconds, and 72°C for 20 seconds, with a final extension of 5 minutes at 72°C. PCR products were separated by electrophoresis through a 2% agarose GelRed (Invitrogen) stained gel for 1 h at 80 V and then visualized using a Gene Genius bio-imaging system.

### RNA extraction, cDNA synthesis and qRT-PCR

RNA was extracted using the RNeasy kit (Qiagen) and reverse transcribed using random hexamers and reverse transcriptase (Superscript III, Invitrogen). Real time PCR was carried out using either Power mix SYBR Green and specific primers (Additional File 5, Table S2) or TaqMan gene expression pre-synthesized reagents and master mix (CD133: Hs01009257_m1 and RPLP0: Hs99999902_m1, Applied Biosystems). Reaction volumes were reduced to 25 μl for SYBR Green and 10 μl for TaqMan, and were carried out as previously described [22]. Gene expression was considered undetectable if fewer than 2/3 reactions were positive after 40 cycles of PCR.

### Flow Cytometry

Live cells were stained with CD133/2(293C)-APC antibody (1:11) (Miltenyi Biotec) and analysed on a CyAn ADP flow cytometer (Dako Cytomation, Denmark). Doublet cells were gated out with pulse width. Dead cells were gated out by DAPI exclusion. More than 250,000 events were analysed.

### Chromatin Immunoprecipitation

ChIP assays were performed as previously described [48]. The antibodies histone H3 (Abcam), rabbit IgG, H3K4me2, and H3K27me3 (Millipore) were used at a 1:100 dilution to immunoprecipitate an equivalent 20 μg of DNA in ChIP assay. To standardize between experiments, the percentage of immunoprecipitation (IP) was calculated by dividing the value of the IP by the value of the corresponding input (both values normalized for dilution factors).

## Competing interests

The authors declare that they have no competing interests.

## Authors' contributions

DP: Conception and design, collection and assembly of data, data analysis and interpretation, manuscript writing. RJP: Collection and assembly of data, data analysis and interpretation. FMF: Data analysis and interpretation, manuscript writing, final approval of the manuscript. EEO, PAB, MCL, MJS and MSS: Provision of study material or patient samples, collection and assembly of data. ATC and NJM: Conception and design, manuscript writing, final approval of the manuscript. All authors read and approved the final manuscript.

## Supplementary Material

Additional file 1**Additional analysis of CD133 promoter methylation in prostate cell lines.** Figure S1: Methylation specific PCR (MSP) analysis of the CD133 promoter with two different primer sets (MSP1 and MSP2) in a panel of prostate cell lines (A). PCR products recognizing methylated (M) and unmethylated (U) CpG sites were analyzed on 2% agarose gels (Positive Control = RC165 DNA methylated in vitro with SssI methylase; Negative Control = water). Pyrosequencing methylation assay of CD133 CpG island in a panel of prostate cell lines (panel B: PYRO 2 assay; panel C: PYRO 3 assay; each bar represents a single CpG site; n = 3 technical replicas;  ± SD; 100% Meth = Methylated human control DNA - QIAGEN; 0% Meth = Unmethylated human control DNA - QIAGEN; 50% Meth = 1:1 mixture of 100% Meth and 0% Meth). (D) Dot-plot showing CD133 promoter methylation (PYRO 1 assay, average of 10 CpG sites) in the same cell lines shown in Figure 1B grouped on the basis of their origin (benign or cancer) and their culture conditions (low FCS = 0%-2%; high FCS = 5%-10%; * = p < 0.05; ** = p < 0.01; *** = p < 0.001 in an unpaired t-test).Click here for file

Additional file 2 **Figure S2: Quantification of expression of the stem cell marker CD133 in prostate cell lines**. Live cells were stained with CD133/2(293C)-APC antibody (Miltenyi Biotec) (CD133-APC) or without any antibody (No Antibody) and analysed by FACs. For each dot plot, X axes: FITC channel fluorescence (not stained); Y axes: APC channel fluorescence (CD133 or No Antibody control); Percent of CD133^+ ^cells is indicated. Click here for file

Additional file 3**Additional analysis of CD133 promoter methylation in prostate primary epithelial cultures and prostate tissues. **Figure S3: MSP analysis of the CD133 promoter with two different primer sets (MSP1 and MSP2) in prostate primary epithelial cultures (A) and in selected populations from primary epithelial cultures (B) (SC = stem cells; TA = transit amplifying cells; CB = committed basal cells; Negative Control = water; NO CELLS = negative control for whole bisulfitome amplification). Pyrosequencing methylation assay of CD133 promoter in prostate tissues (panel C: PYRO 2 assay; panel D: PYRO 3 assay; DNA extracted from glass slides of snap frozen tissue sections which were pooled together) and in prostate primary epithelial cultures (panel E: PYRO 2 assay; each bar represent a single CpG site; n = 3 technical replicas;  ± SD).Click here for file

Additional file 4 **Table S1: List of the cell lines used**. Table indicating the cell lines used in this manuscript, their origin and culture conditions. Click here for file

Additional file 5 **Table S2: List of the primers used Table indicating the primers used in this manuscript for PCR amplification and pyrosequencing**. Click here for file

Additional file 6 **DNA methylation analysis of CD133 promoter using pyrosequencing.** Figure S4: (A) Diagram depicting a typical histogram generated by pyrosequencing methylation analysis of CD133 promoter with PYRO 1 assay and the position of each CpG site on the genomic sequence (Exon 1 A is shown in yellow). (B-C) Example of typical pyrograms generated with PYRO 1 assay using human (B) or mouse (C) bisulfite converted DNA as a template for PCR. No PCR product or sequence was generated with mouse DNA Click here for file
