# Supplementary figures and images for: Regulation of the stem cell marker CD133 is independent of promoter hypermethylation in human epithelial differentiation and cancer
Source: Mol Cancer. 2011 Jul 29;10:94. doi: 10.1186/1476-4598-10-94 (PMC3162587; doi:10.1186/1476-4598-10-94)

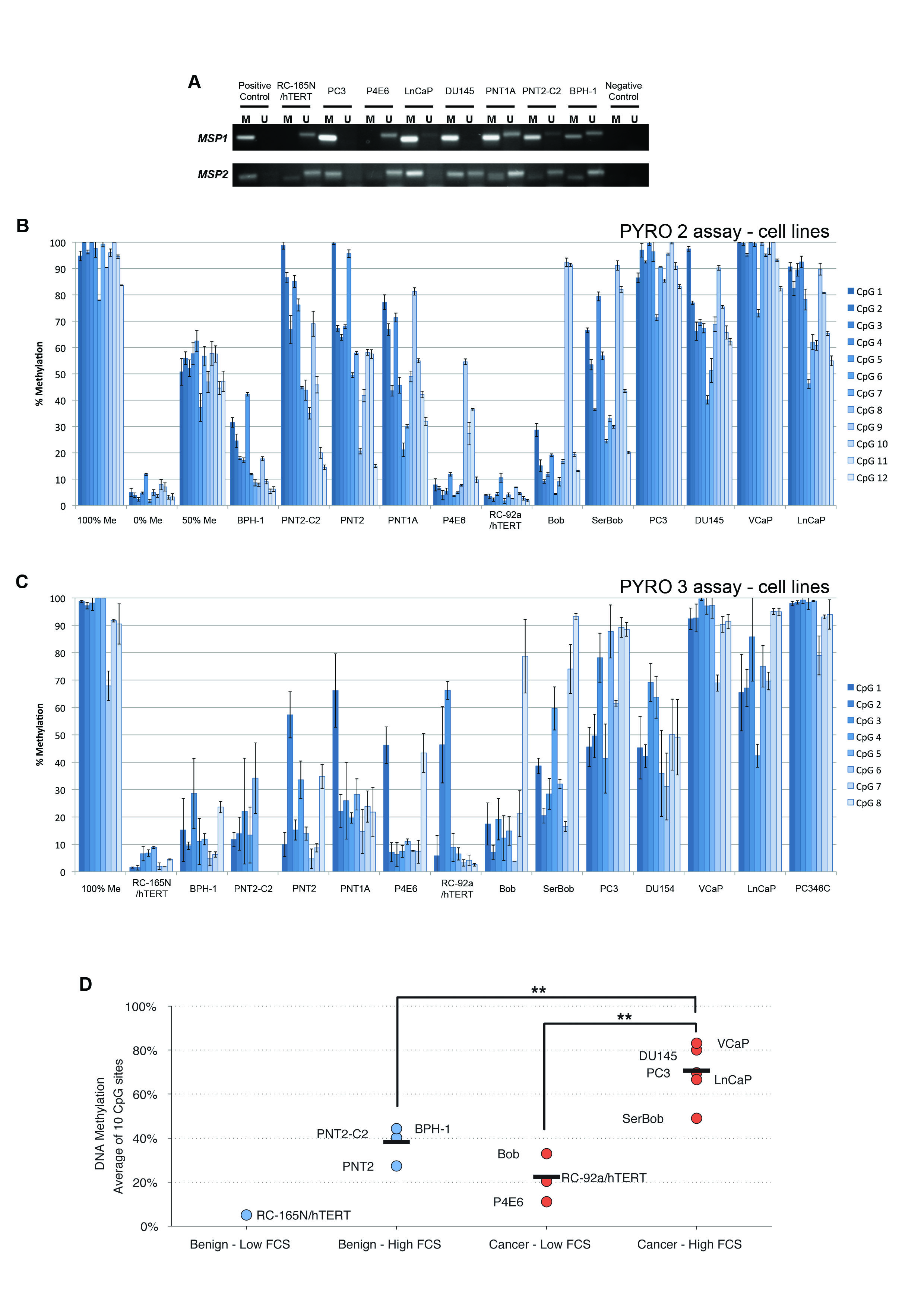

Supplement: Additional file 1 — Additional analysis of CD133 promoter methylation in prostate cell lines. Figure S1: Methylation specific PCR (MSP) analysis of the CD133 promoter with two different primer sets (MSP1 and MSP2) in a panel of prostate cell lines (A). PCR products recognizing methylated (M) and unmethylated (U) CpG sites were analyzed on 2% agarose gels (Positive Control = RC165 DNA methylated in vitro with SssI methylase; Negative Control = water). Pyrosequencing methylation assay of CD133 CpG island in a panel of prostate cell lines (panel B: PYRO 2 assay; panel C: PYRO 3 assay; each bar represents a single CpG site; n = 3 technical replicas; ± SD; 100% Meth = Methylated human control DNA - QIAGEN; 0% Meth = Unmethylated human control DNA - QIAGEN; 50% Meth = 1:1 mixture of 100% Meth and 0% Meth). (D) Dot-plot showing CD133 promoter methylation (PYRO 1 assay, average of 10 CpG sites) in the same cell lines shown in Figure 1B grouped on the basis of their origin (benign or cancer) and their culture conditions (low FCS = 0%-2%; high FCS = 5%-10%; * = p < 0.05; ** = p < 0.01; *** = p < 0.001 in an unpaired t-test). [file 1476-4598-10-94-S1.JPEG]

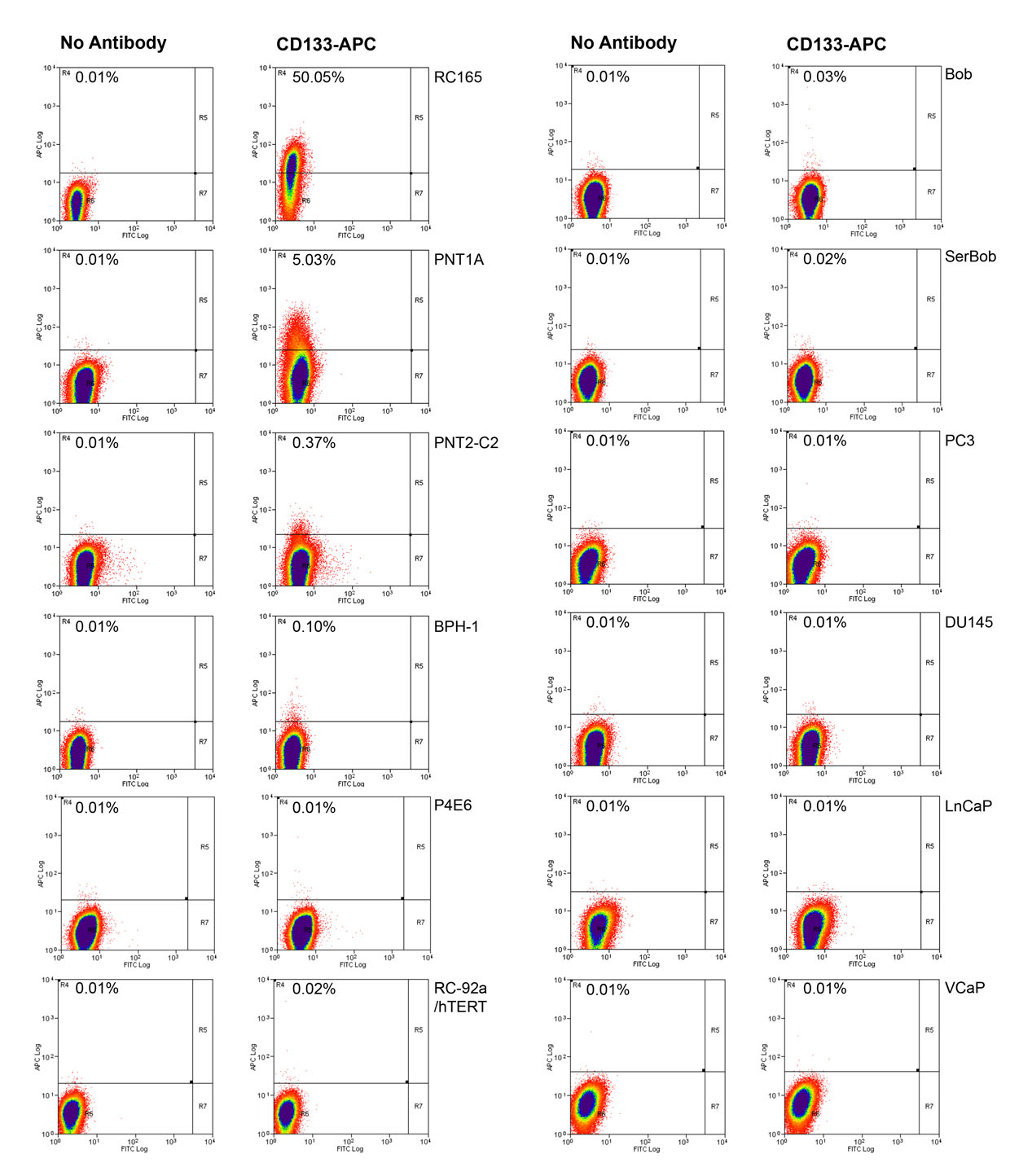

Supplement: Additional file 2 — Figure S2: Quantification of expression of the stem cell marker CD133 in prostate cell lines. Live cells were stained with CD133/2(293C)-APC antibody (Miltenyi Biotec) (CD133-APC) or without any antibody (No Antibody) and analysed by FACs. For each dot plot, X axes: FITC channel fluorescence (not stained); Y axes: APC channel fluorescence (CD133 or No Antibody control); Percent of CD133+ cells is indicated. [file 1476-4598-10-94-S2.JPEG]

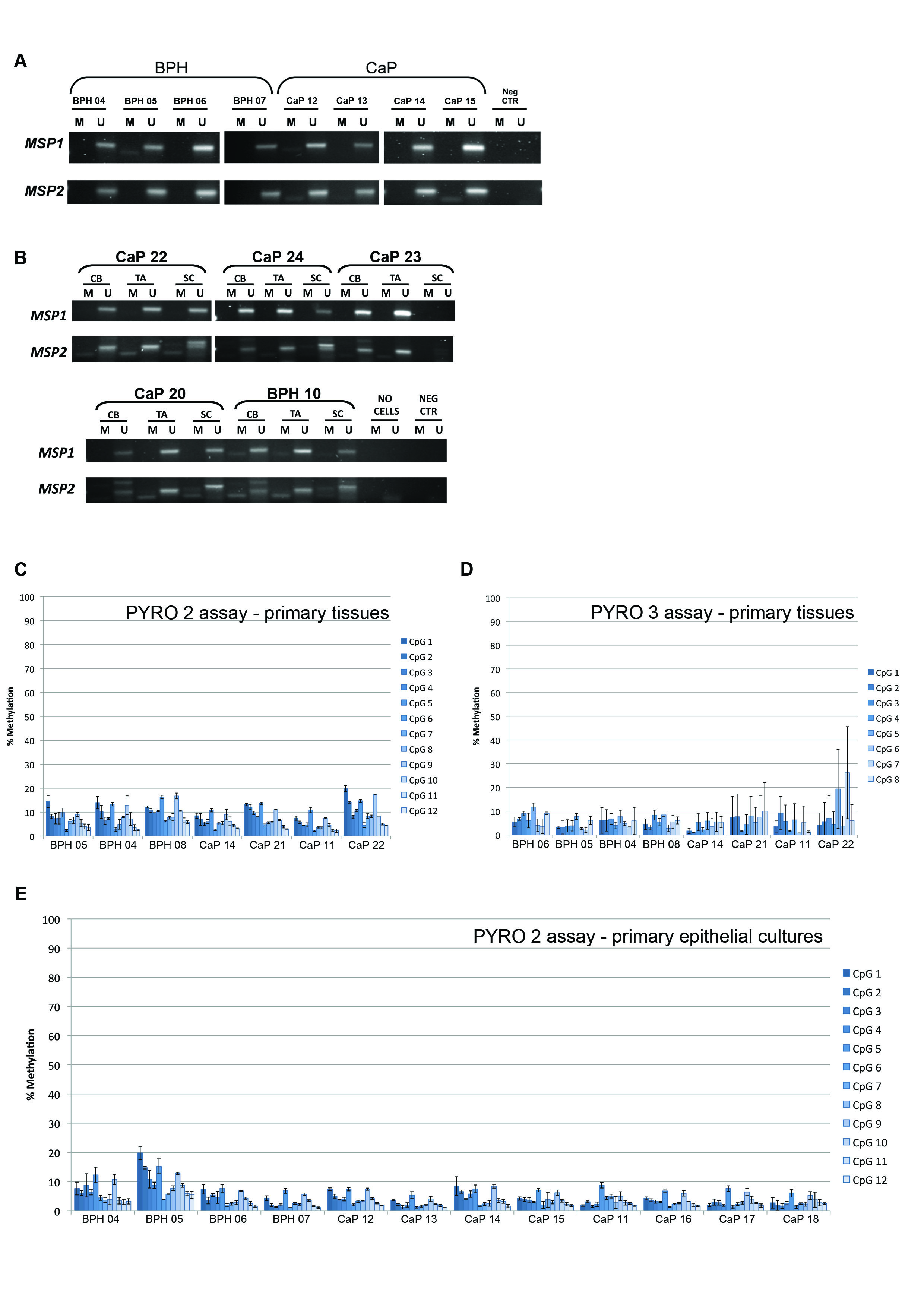

Supplement: Additional file 3 — Additional analysis of CD133 promoter methylation in prostate primary epithelial cultures and prostate tissues. Figure S3: MSP analysis of the CD133 promoter with two different primer sets (MSP1 and MSP2) in prostate primary epithelial cultures (A) and in selected populations from primary epithelial cultures (B) (SC = stem cells; TA = transit amplifying cells; CB = committed basal cells; Negative Control = water; NO CELLS = negative control for whole bisulfitome amplification). Pyrosequencing methylation assay of CD133 promoter in prostate tissues (panel C: PYRO 2 assay; panel D: PYRO 3 assay; DNA extracted from glass slides of snap frozen tissue sections which were pooled together) and in prostate primary epithelial cultures (panel E: PYRO 2 assay; each bar represent a single CpG site; n = 3 technical replicas; ± SD). [file 1476-4598-10-94-S3.JPEG]

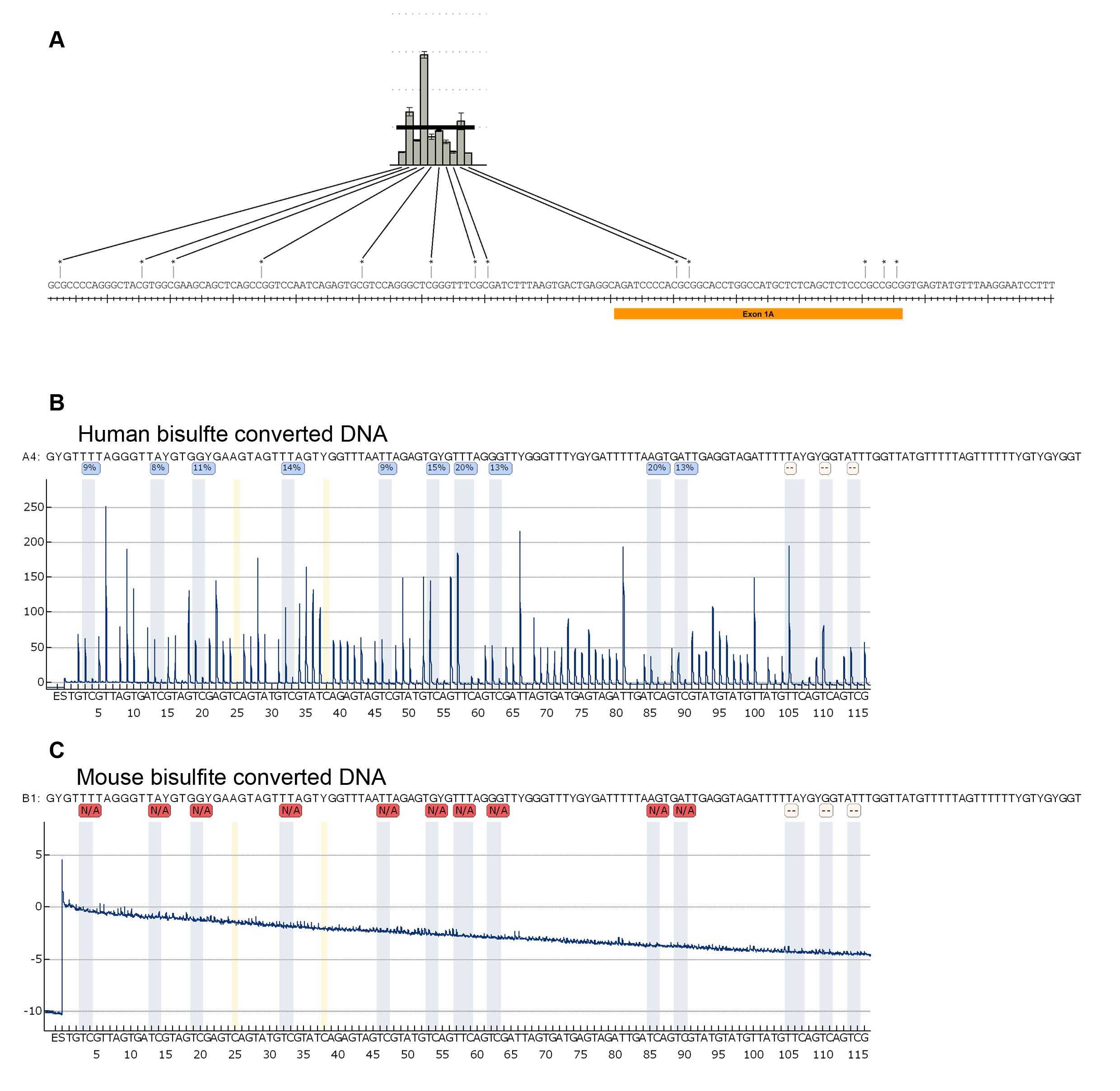

Supplement: Additional file 6 — DNA methylation analysis of CD133 promoter using pyrosequencing. Figure S4: (A) Diagram depicting a typical histogram generated by pyrosequencing methylation analysis of CD133 promoter with PYRO 1 assay and the position of each CpG site on the genomic sequence (Exon 1 A is shown in yellow). (B-C) Example of typical pyrograms generated with PYRO 1 assay using human (B) or mouse (C) bisulfite converted DNA as a template for PCR. No PCR product or sequence was generated with mouse DNA [file 1476-4598-10-94-S6.JPEG]
