# Supplementary material for: Regulation of the stem cell marker CD133 is independent of promoter hypermethylation in human epithelial differentiation and cancer
Source: Mol Cancer. 2011 Jul 29;10:94. doi: 10.1186/1476-4598-10-94 (PMC3162587; doi:10.1186/1476-4598-10-94)
Supplement: Additional file 4 — Table S1: List of the cell lines used. Table indicating the cell lines used in this manuscript, their origin and culture conditions. [file 1476-4598-10-94-S4.PDF]

List of cell lines used:

Cell line	Origin	Culture Media	Reference
RC-165N/hTERT	obtained with kind permission from Johng Rhim	KSFM medium with supplements (bovine pituitary extract 50μg/ml and human recombinant epidermal growth factor 5ng/ml)	(Miki et al, 2007)
RC-92a/hTERT	obtained with kind permission from Johng Rhim	KSFM medium with supplements (bovine pituitary extract 50μg/ml and human recombinant epidermal growth factor 5ng/ml)	(Miki et al, 2007)
Bob	obtained with kind permission from David Hudson	KSFM with epidermal growth factor, bovine pituitary extract (Invitrogen), leukemia inhibitory factor (Chemicon, Chandlers Ford, UK), cholera toxin (Sigma, Poole UK), granulocyte macrophage colony stimulating factor, stem cell factor (First Link (UK) Ltd., Birmingham, UK)	(Attard et al, 2009)
SerBob	obtained with kind permission from David Hudson	KSFM with epidermal growth factor, bovine pituitary extract (Invitrogen), leukemia inhibitory factor (Chemicon, Chandlers Ford, UK), cholera toxin (Sigma, Poole UK), granulocyte macrophage colony stimulating factor, stem cell factor (First Link (UK) Ltd., Birmingham, UK) and 10% FCS	(Attard et al, 2009)
PNT2	ECACC	RPMI with 10% FCS	
PNT2-C2	Clone derived in York	RPMI with 10% FCS	
PNT1A	obtained with kind permission from P Berthon	RPMI with 10% FCS	
BPH-1	obtained with kind permission from Simon Hayward	RPMI with 5% FCS	(Hayward et al, 1995)
P4E6	Derived in York	KSFM medium with supplements (bovine pituitary extract 50μg/ml and human recombinant epidermal growth factor 5ng/ml) and 2% FCS	(Maitland et al, 2001)
PC3	ECACC	Hams-F12 medium with 7% FCS	
DU154	ATCC	RPMI with 10% FCS	
LnCaP	ECACC	RPMI with 10% FCS	
VCaP	ATCC	RPMI with 10% FCS	
PC346C	obtained with kind permission from Chris Bangma	As described in Dubbink et al., 1996	(Dubbink et al, 1996)

References:

Attard, G, Rizzo, S, Ledaki, I, Clark, J, Reid, AH, Thompson, A, Khoo, V, de Bono, JS, Cooper, CS and Hudson, DL. (2009) A novel, spontaneously immortalized, human prostate cancer cell line, Bob, offers a unique model for pre-clinical prostate cancer studies. *Prostate*, **69**: 1507-1520.

Dubbink, HJ, Verkaik, NS, Faber, PW, Trapman, J, Schroder, FH and Romijn, JC. (1996) Tissue specific and androgen-regulated expression of human prostate-specific transglutaminase. *Biochem J*, **315 (Pt 3)**: 901-908.

Hayward, SW, Dahiya, R, Cunha, GR, Bartek, J, Deshpande, N and Narayan, P. (1995) Establishment and characterization of an immortalized but non-transformed human prostate epithelial cell line: BPH-1. *In Vitro Cell Dev Biol Anim*, **31**: 14-24.

Maitland, NJ, Macintosh, CA, Hall, J, Sharrard, M, Quinn, G and Lang, S. (2001) In vitro models to study cellular differentiation and function in human prostate cancers. *Radiat Res*, **155**: 133-142.

Miki, J, Furusato, B, Li, H, Gu, Y, Takahashi, H, Egawa, S, Sesterhenn, IA, McLeod, DG, Srivastava, S and Rhim, JS. (2007) Identification of putative stem cell markers, CD133 and CXCR4, in hTERT-immortalized primary nonmalignant and malignant tumor-derived human prostate epithelial cell lines and in prostate cancer specimens. *Cancer Res*, **67**: 3153-3161.
