# Supplementary material for: Regulation of the stem cell marker CD133 is independent of promoter hypermethylation in human epithelial differentiation and cancer
Source: Mol Cancer. 2011 Jul 29;10:94. doi: 10.1186/1476-4598-10-94 (PMC3162587; doi:10.1186/1476-4598-10-94)
Supplement: Additional file 5 — Table S2: List of the primers used Table indicating the primers used in this manuscript for PCR amplification and pyrosequencing. [file 1476-4598-10-94-S5.PDF]

Pyrosequencing

Name	Sequence (5' to 3')	Length
PYRO 1 DIR	GAGTAGGTATTTTTATAGGAAATGGATG	28
PYRO 1 REV	Biotin-AATAAAAAAAAATTCCTTAAACATACTCA	28
PYRO 1 SEQ	TTGTTTAGGTGTTGGTGG	18
PYRO 2 DIR	TTAGGTTTTAGTTAGGGATAGAGGAAGT	28
PYRO 2 REV	Biotin-CCAAACCCTAACTCCTAAATTATTTATAAC	30
PYRO 2 Seq	TTTGTTTTTAGTTAAGTTAGAGGGT	25
PYRO 3 DIR	GGTTGGGGGTTAGAGGAATT	20
PYRO 3 REV	Biotin-CCCCATCCCCAATAAATAA	19
PYRO 3 Seq	GGGGTTAGAGGAATTG	16

ChIP-qPCR

Name	Sequence (5' to 3')	Length
ChIP 1 DIR	TATGGCTTTATGCTGTTTTTCAA	23
ChIP 1 REV	CTCATCCCGGCCGCATTAGAC	21
ChIP 2 DIR	CGCCGCGGTGAGTATGTTTA	20
ChIP 2 REV	GACGTGGCGGGAGGCAGGAG	20
ChIP 3 DIR	CCAGAAGCCGGGTCATAAATAAT	23
ChIP 3 REV	AGCGAACCCGTCCACTCCTCACT	23
ChIP 4 DIR	GAACTGCGGGGAGAGCGTGGTG	22
ChIP 4 REV	TCCCCGAGAGCGAGTCCGAAGTC	23
ChIP 5 DIR	CGACCACAGCGGGAGTAG	18
ChIP 5 REV	GCGAGAGGCTGGGAAGGT	18
ChIP 6 DIR	CCGCCCCGCCGCTCATTC	18
ChIP 6 REV	GCTTCCCCGCCCTTTACCTC	20
ChIP 7 DIR	TGCGAACCCTATGCGAAATCC	21
ChIP 7 REV	TAAAGGGCACTGCTGAATAGACA	23
ChIP 8 DIR	TTGCTGGAATGAATTAGATACTGT	24
ChIP 8 REV	CTGAGGCGCCCAAACTGTGT	20

Methylation Specific PCR

Name	Sequence (5' to 3')	Length
MSP1-M-dir	GGCGGTTTTATATTTAGGTTTTCGTTC	27
MSP1-M-rev	CGAACCCTCGAACGTAACG	19
MSP1-U-dir	TTATTATGGTGGTTTTATATTTAGGTTTTTGTT TG	35
MSP1-U-rev	ACTACAACCAAACCCTCAAACATAACA	27
MSP2-M-dir	GTGGTGGTGTCGGTGTTC	18
MSP2-M-rev	AATAAATAAAAAAAAAACCCAAACGAAACGT CG	33
MSP2-U-dir	AGTGTGGTGGTGTTGGTGTTTG	22
MSP2-U-rev	CAATAAATAAAAAAAAAACCCAAACAAAACAT CA	34

qRT-PCR (SYBR Green)

Name	Sequence (5' to 3')	Length
qPCR-CD133-DIR	TTGCGGTAAAACTGGCTAAG	20
qPCR-CD133-REV	TGGGCTTGTCATAACAGGAT	20
qPCR-HPRT-Dir	CCAAATCCTCAGCATAATGATTAGG	25
qPCR-HPRT-Rev	GATGATGAACCAGGTTATGACC	22
